# Ischemic Colitis Associated With Tirzepatide Therapy in a Young Female Patient: A Case Report

**DOI:** 10.7759/cureus.101437

**Published:** 2026-01-13

**Authors:** Simone Sekhon, Ibaadat Kahlon, William Latson

**Affiliations:** 1 Gastroenterology, Saint Agnes Medical Center, Fresno, USA; 2 Internal Medicine, Saint Agnes Medical Center, Fresno, USA

**Keywords:** drug-induced colitis, gastrointestinal bleeding, ischemic colitis, tirzepatide, weight loss and obesity

## Abstract

Tirzepatide has been approved for the management of obesity, obstructive sleep apnea, and type 2 diabetes mellitus; while gastrointestinal side effects are commonly seen, ischemic colitis has been only rarely described. We present the case of a 32-year-old woman with no prior medical history who developed hematochezia and abdominal pain after six weeks of tirzepatide therapy for weight loss. Laboratory evaluation showed neutrophilic leukocytosis without anemia or electrolyte abnormalities, and stool studies were negative for infection. A CT of the abdomen and pelvis excluded obstruction or wall thickening but incidentally revealed hepatic steatosis and adnexal lesions. Colonoscopy demonstrated patchy erosions, erythema, and ulcerations in the sigmoid and descending colon, with biopsy confirming ischemic colitis. She was managed conservatively with bowel rest, intravenous fluids, and close monitoring, with stable hemoglobin and complete resolution of symptoms. Although ischemic colitis is more typically linked to vascular disease, hypotension, or vasoconstrictive drugs, recent reports have suggested an association with glucagon-like peptide-1 (GLP-1) receptor agonists, including semaglutide and tirzepatide. The temporal relationship with tirzepatide initiation, absence of infection, and confirmatory histology strongly support drug-induced ischemia in this case, underscoring the importance of clinician awareness of this rare but significant complication in patients presenting with rectal bleeding or abdominal pain while on incretin-based therapy.

## Introduction

Tirzepatide is a novel dual glucose-dependent insulinotropic polypeptide (GIP) and glucagon-like peptide-1 (GLP-1) receptor agonist that enhances glucose-dependent insulin secretion, delays gastric emptying, and promotes satiety, leading to clinically meaningful weight reduction. Its approval for obesity management followed large randomized clinical trials demonstrating significant and sustained weight loss compared with placebo, alongside improvements in metabolic parameters. While gastrointestinal adverse events such as nausea, vomiting, diarrhea, and constipation are the most frequently reported, emerging post-marketing data continue to expand the recognized safety profile of the drug [1]. 

Ischemic colitis is typically seen in older adults with vascular risk factors and is uncommon in otherwise healthy young patients. Medication-associated ischemic colitis has been reported with several agents, and rare reports have begun to describe possible associations with incretin-based therapies; however, evidence remains limited.

The objective of this case report is to describe a rare presentation of ischemic colitis occurring in temporal association with tirzepatide therapy and to discuss the diagnostic evaluation and clinical management considerations.

## Case presentation

A previously healthy 32-year-old woman presented with several days of crampy lower abdominal discomfort, followed by the sudden onset of rectal bleeding. She reported a history of chronic loose stools but denied recent travel, antibiotic use, nonsteroidal anti-inflammatory drugs, or anticoagulants. Six weeks before admission, she had begun weekly tirzepatide injections for weight reduction.

On arrival, her vital signs were within normal limits: blood pressure 118/82 mmHg, heart rate 74 beats per minute (bpm), respiratory rate 18 breaths per minute, temperature 36.9°C, and oxygen saturation 100% on room air. Physical examination demonstrated a soft, non-distended abdomen and fresh blood on digital rectal examination.

Laboratory studies revealed leukocytosis with neutrophil predominance, hemoglobin of 13.7 g/dL, and normal kidney and liver function (Table 1).

**Table 1:** Complete blood count (CBC) and comprehensive metabolic panel (CMP) results

Test	Result	Reference Range
White Blood Cell Count	13.4 ×10³/µL	4.0–10.0 ×10³/µL
Neutrophils (Percentage)	78%	40–70%
Hemoglobin	13.7 g/dL	12.0–16.0 g/dL
Hematocrit	41%	36–46%
Red Blood Cell Count	4.7 ×10⁶/µL	4.2–5.4 ×10⁶/µL
Platelet Count	260 ×10³/µL	150–400 ×10³/µL
Mean Corpuscular Volume	88 fL	80–100 fL
Mean Corpuscular Hemoglobin Concentration	33 g/dL	32–36 g/dL
Red Cell Distribution Width	12.50%	11–15%
Sodium	139 mmol/L	135–145 mmol/L
Potassium	4.1 mmol/L	3.5–5.0 mmol/L
Chloride	103 mmol/L	98–106 mmol/L
Carbon Dioxide (Bicarbonate)	25 mmol/L	22–29 mmol/L
Blood Urea Nitrogen	14 mg/dL	7–20 mg/dL
Creatinine	0.9 mg/dL	0.6–1.3 mg/dL
Glucose	92 mg/dL	70–99 mg/dL
Calcium	9.4 mg/dL	8.5–10.5 mg/dL
Total Protein	7.2 g/dL	6.0–8.3 g/dL
Albumin	4.3 g/dL	3.5–5.0 g/dL
Aspartate Aminotransferase	22 U/L	10–40 U/L
Alanine Aminotransferase	24 U/L	7–56 U/L
Alkaline Phosphatase	88 U/L	44–147 U/L
Total Bilirubin	0.7 mg/dL	0.1–1.2 mg/dL

Cross-sectional imaging of the abdomen and pelvis showed no acute colonic pathology, though hepatomegaly with fatty infiltration, a uterine fibroid, and a right adnexal cyst were noted incidentally.

**Figure 1:**
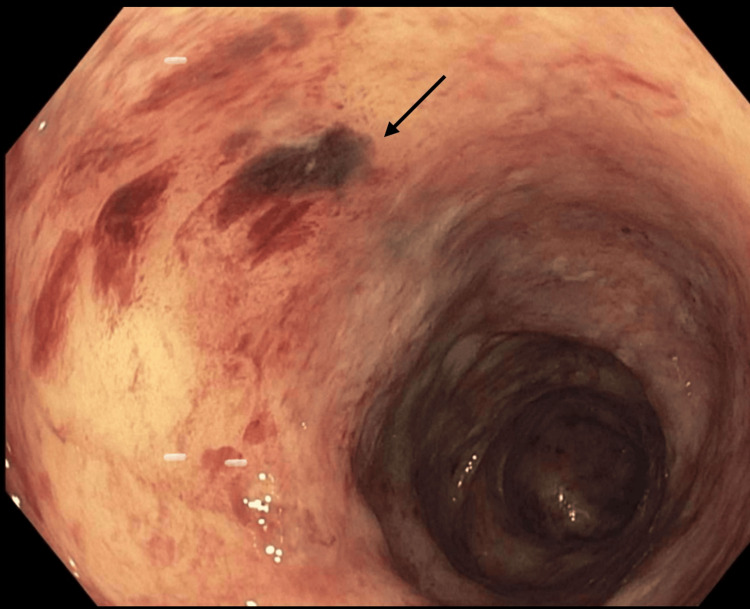
Colonoscopy revealed patchy erythema, superficial ulcerations, and erosions as demonstrated by the black arrow.

Colonoscopy identified patchy erythema, superficial ulcerations, and erosions extending through the sigmoid and descending colon, with normal findings in the rectum and terminal ileum (Figure 1).

**Figure 2:**
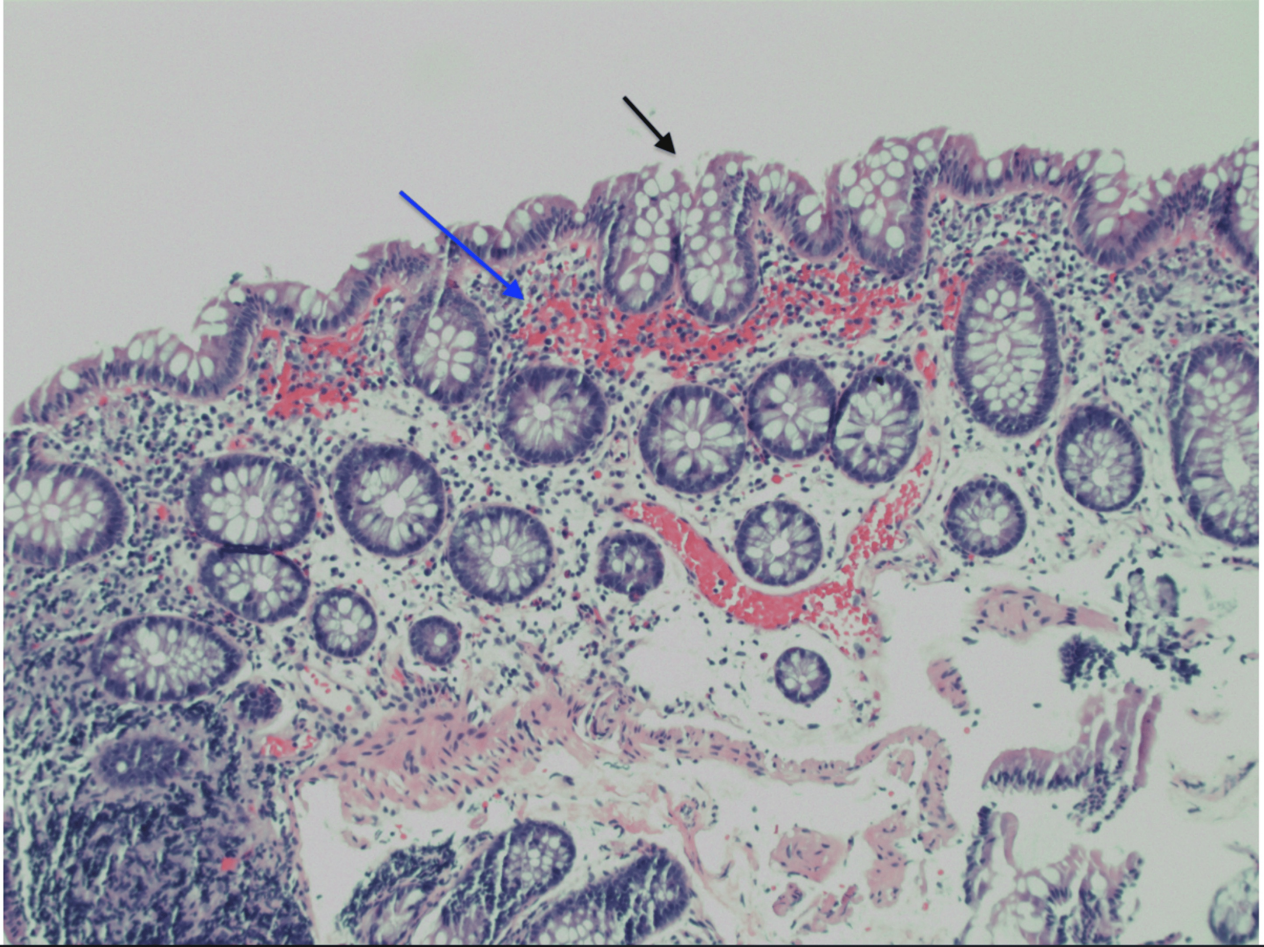
Colonic mucosa with surface epithelial injury (black arrow) and lamina propria neutrophilic infiltrate (blue arrow) (hematoxylin and eosin stain, original magnification ×100).

Biopsies from the descending colon demonstrated crypt injury and ulceration in keeping with ischemic colitis (Figure 2), whereas rectal samples showed only benign mucosa. Stool analyses for enteric pathogens, parasites, and *Clostridioides difficile* were negative.

She was treated conservatively with intravenous hydration, bowel rest, and close hemoglobin monitoring. Her bleeding and abdominal pain resolved within 48 hours. She was discharged in stable condition with advice to discontinue tirzepatide and continue gastroenterology follow-up.

## Discussion

Tirzepatide-associated ischemic colitis was recently reported by Bayless et al. in a 62-year-old woman who developed ischemic colitis while on tirzepatide for weight loss [2]. Similarly, semaglutide has been implicated in ischemic colitis in patients without any vascular risk factors [3].

The mechanism by which these therapies may induce ischemic injury is hypothetical. Wichelmann et al. suggested that delayed gastric emptying and altered splanchnic perfusion could predispose to colonic hypoperfusion [4]. Another well-recognized side effect of these medications is constipation, which can impair mucosal blood flow with increased intramural pressure. The exact mechanism still remains unknown. Wright et al. further highlighted gastrointestinal adverse effects with dual GLP-1/GIP agonists [5]. 

Our case is notable for occurring in a young patient without any risk factors for ischemic colitis. The temporal relationship (symptom onset within one to two months of tirzepatide initiation), combined with exclusion of infection and confirmation of ischemia histologically, supports a likely causal association. Huang et al. found that most tirzepatide adverse events occur within the first six months of therapy, consistent with our case [6]. Schoenfeld et al. also underscored the importance of continued monitoring for adverse effects during long-term tirzepatide use [7]. Using the Hartwig and Siegel adverse drug reaction severity assessment scale, this event was classified as moderate level 4, as it required hospitalization, diagnostic evaluation, and discontinuation of tirzepatide.

Clinicians should recognize ischemic colitis as a possible, though rare, adverse effect of GLP-1/GIP agonists. Pharmacovigilance databases and real-world observational studies [8-10], as well as recent commentary in the British Medical Journal (BMJ) [11], suggest that the adverse event spectrum may be broader than initially reported in trials. Our case underscores the need for heightened awareness and prompt discontinuation of the offending agent in patients presenting with hematochezia or abdominal pain.

This report is limited by its single-patient design, which precludes establishing causality or generalizing findings to broader populations. A temporal association between tirzepatide exposure and symptom onset was observed. Long-term outcomes beyond the acute episode were not assessed, and the precise mechanism underlying the ischemic injury could not be determined. Additional case series and controlled studies are needed to better clarify potential associations between tirzepatide and ischemic colitis.

## Conclusions

This case describes an episode of ischemic colitis occurring in temporal association with tirzepatide use in a previously healthy young woman without traditional predisposing factors. The presentation with abdominal pain and hematochezia, together with colonoscopic and histopathologic evidence of ischemia, highlights that this condition may occur even in patients considered low risk.

The case underscores the importance of considering ischemic colitis in the differential diagnosis of new-onset rectal bleeding or abdominal pain in patients receiving incretin-based therapies and illustrates that symptoms may be nonspecific early in the course. Clinical improvement with supportive care and drug discontinuation was observed.
